# Non-invasive Ventilation for Pediatric Hypoxic Acute Respiratory Failure Using a Simple Anesthetic Mask With 3D Printed Adaptor: A Case Report

**DOI:** 10.3389/fped.2021.710829

**Published:** 2021-08-24

**Authors:** Gerrit J. Muller, Renee Hovenier, Jip Spijker, Monica van Gestel, Rozalinde Klein-Blommert, Dick Markhorst, Coen Dijkman, Reinout A. Bem

**Affiliations:** ^1^Pediatric Intensive Care Unit, Emma Children's Hospital, Amsterdam University Medical Centers, Amsterdam, Netherlands; ^2^Technical Medicine, University of Twente, Enschede, Netherlands; ^3^Industrial Design Engineering, Technical University of Delft, Delft, Netherlands; ^4^Department for Medical Innovation and Development, Amsterdam University Medical Centers, Amsterdam, Netherlands

**Keywords:** children, case report, non-invasive ventilation, acute respiratory failure, respiratory support, 3D printing

## Abstract

Non-invasive ventilation (NIV) is increasingly used in the supportive treatment of acute respiratory failure in children in the pediatric intensive care unit (PICU). However, finding an optimal fitting commercial available NIV face mask is one of the major challenges in daily practice, in particular for young children and those with specific facial features. Large air leaks and pressure-related skin injury due to suboptimal fit are important complications associated with NIV failure. Here, we describe a case of a 4-year old boy with cardiofaciocutaneous syndrome and rhinovirus-associated hypoxic acute respiratory failure who was successfully supported with NIV delivered by a simple anesthetic mask connected to a headgear by an in-house developed and 3D printed adaptor. This case is an example of the clinical challenge related to pediatric NIV masks in the PICU, but also shows the potential of alternative NIV interfaces e.g., by using a widely available and relatively cheap simple anesthetic mask. Further personalized strategies (e.g., by using 3D scanning and printing techniques) that optimize NIV mask fitting in children are warranted.

## Introduction

In the past two decades, there has been an increase in the use of non-invasive ventilation (NIV) for acute respiratory failure (ARF) in critically-ill children (1–5). Although NIV can avoid invasive endotracheal intubation in many cases, the failure rate associated with NIV in children may be high, e.g., up to 30% in children with hypoxic ARF (6). One of the major challenges in pediatric NIV is the fitting of the mask interface, most commonly a total face- (covering eyes, nose and mouth), full face/oronasal- (covering mouth and nose) or nasal mask (3). Improper fit of the mask may lead to serious air leaks as well as pressure-related skin injury, culminating in inefficient respiratory support and patient discomfort. In daily clinical practice, this is a problem in particular for young children, due to the limited availability of different commercial (non-vented) mask sizes and designs. Finding a NIV interface that addresses the great variation in face shapes and contours of growing children, as well as patients with specific features as part of a (genetic) syndrome, remains a major challenge.

**Figure 1:**
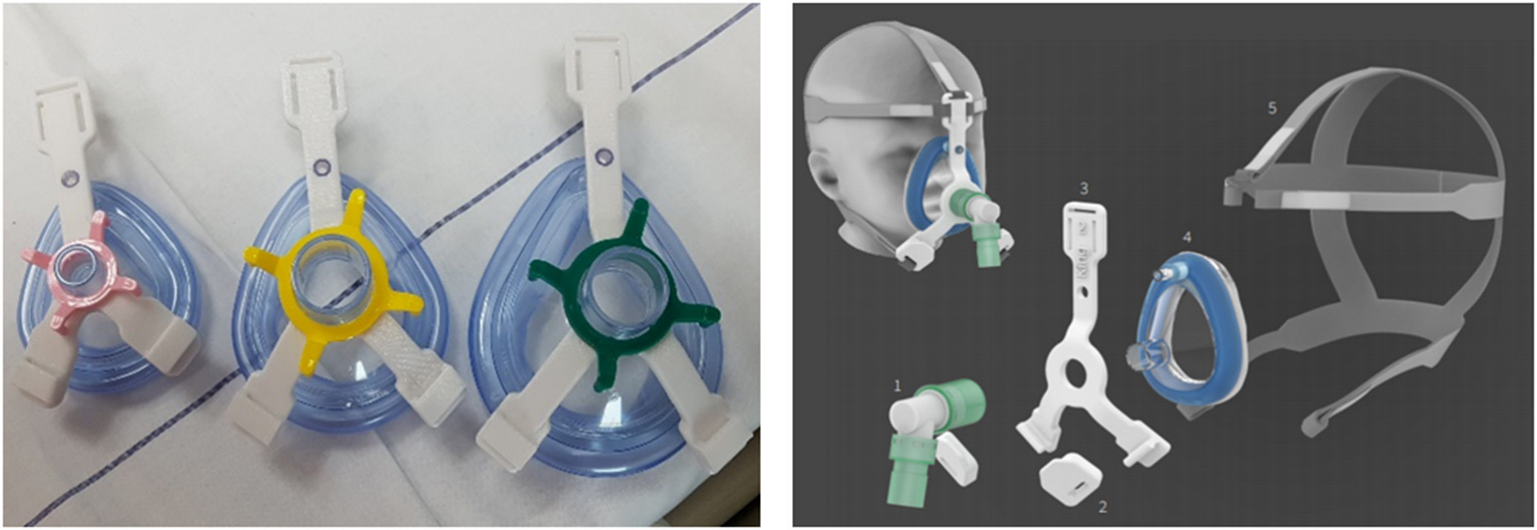
In-house developed NIV interface adaptor. An in-house 3D printed adaptor [left panel, biocompatible polycarbonate (PC), compliant with ISO 10993 and USP Class VI, white three-lead adaptor in different sizes] to connect a simple anesthetic mask to a five-point head gear for NIV application in children [right panel, 1. ventilator tubing and swivel; 2. 3D printed PC-ISO quick-release clips; 3. 3D printed PC-ISO adaptor; 4. simple anesthetic mask; 5. five-point head gear (Respireo SOFT child, VitalAire)].

Stimulated by this challenge in pediatric NIV, and also fueled by current scarcity in ventilator equipment due to the COVID-19 pandemic, we have recently developed an in-house 3D printed adaptor. This re-usable and quick-release adaptor connects a simple anesthetic mask to a five-point headgear (Figure 1). In the last year, we have successfully managed patients in our pediatric intensive care unit (PICU) with this alternative NIV interface, in rotation with commercial available non-vented pediatric NIV masks. Here, as an example, we report its use in a case of a 4-year old boy with cardiofaciocutaneous syndrome admitted to our PICU for rhinovirus-induced hypoxic ARF (see Supplemental File for CARE checklist).

## Case Description

The 4-year old boy had previously been diagnosed with cardiofaciocutaneous syndrome (BRAF mutation) with developmental disability, muscle weakness, obstructive (laryngeal malacia) and central sleep apnea, severe visual impairment, small atrial septum defect, epilepsy, and recurrent respiratory infections. As part of the underlying syndrome he had noticeable coarse features with a broad face with ocular hypertelorism and low-set ears. On the day of hospital admission, the patient experienced dyspnea with mucus hypersecretion without fever, and need for oxygen supplementation as determined by lower bound oxygen saturation (SpO_2_) levels of 77–92% monitored in the home-setting. There were no signs of convulsions. He was transported from home to the emergency room with a non-rebreather oxygen mask at 15 L/min 100% oxygen by emergency ambulance. His airway was partially obstructed by mucus and saliva, requiring frequent suctioning. Despite 100% oxygen therapy by a non-rebreather mask and later-on high flow nasal cannula at 30L/min his SpO_2_ levels remained at 85–93%, with inefficient and rapid (up to 38 times per minute) breathing and a heart rate up to 130 times per minute.

Once admitted to the PICU he was treated with NIV, using a Hamilton C6 ventilator in the NIV-spontaneous/timed (ST) mode, for persistent hypoxic ARF. With respect to the severity of the patient's underlying illness, both the team of PICU physicians as well as the parents expressed the preference to make a strong effort to attempt to avoid endotracheal intubation, as a prolonged and complicated invasive mechanical ventilation trajectory could be expected. A rhinovirus-associated lower respiratory tract infection was diagnosed as determined by multiplex respiratory virus PCR test of a nasopharyngeal sample. He received intravenous antibiotics (amoxicillin with clavulanic acid) to treat a possible bacterial respiratory co-infection, and nebulization with hypertonic saline and dornase alfa with physiotherapy to support mucus clearance.

**Figure 2:**
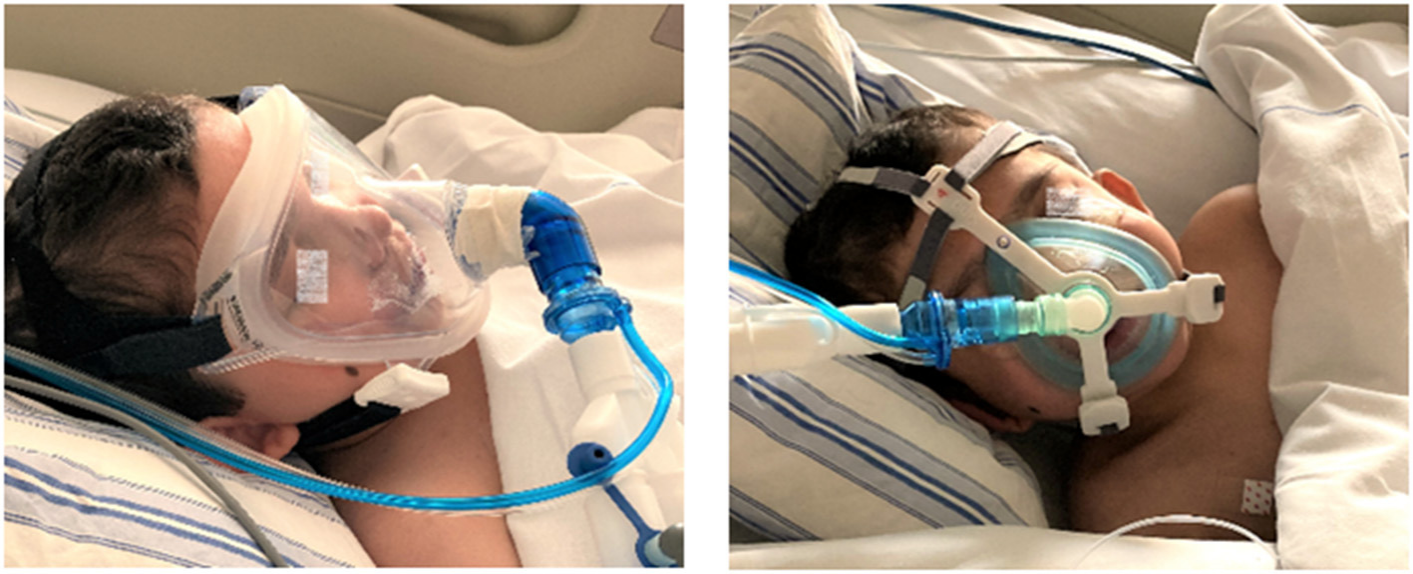
NIV interface application in the child. NIV provided by a total face mask (left panel) and by a simple anesthetic mask using an in-house 3D printed adaptor for connection to a five-point head gear (right panel). Photographs taken and printed with permission granted by the parents.

**Figure 3:**
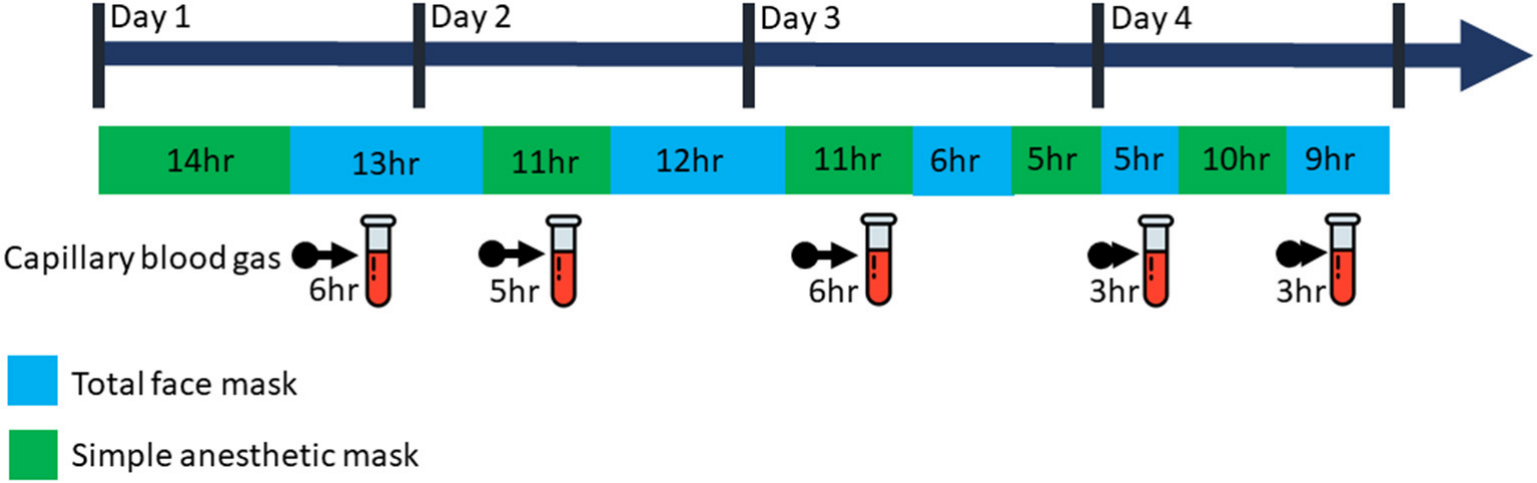
Timeline of switches between NIV interfaces. Timeline for the relatively stable four-day period (relating to Table 1) showing the frequency of switches between the total face mask and simple anesthetic mask, as per decision of the attending nurse and/or physician (approximate time in hours spend on each mask as indicated in the figure). Switches between masks was timed with moments of patient (airway) care and sleep/awake cycles as much as possible. In addition, decision to switch masks was based on clinical judgement (e.g., increased respiratory distress, anxiety). To determine patient ventilation, pCO2 values were measured in capillary blood gases, which were taken daily, as per decision of the attending physician and in timing with normal patient care and sleep/awake cycles.

During the stay at our PICU, NIV was provided with the need to rotate (1–3 times per day) two different face masks: a total face mask covering the mouth, nose and eyes (Respironics PerforMax® full face mask size small, Phillips, the Netherlands) and a simple anesthetic mask (Ambu®King Mask, size 4, Denmark) connected to a five-point head gear by our in-house 3D printed reusable adaptor (Figures 1, 2). Support with a nasal mask (MiniMe®, Sleepnet Corporation, Hampton, USA) was also attempted on two occasions, but was not continued as it was associated with large air leaks up to 90% through the mouth leading to many ventilator alarms and increased need of oxygen. Table 1 shows the air leaks, and levels of hypercapnia/hypoxemia during the treatment with the total face- and simple anesthetic mask as measured on 15 random timepoints and daily capillary blood gas analysis during a relatively stable 4-day period in the acute phase of hypoxic ARF (first week of admission) (see Figure 3 for timeline of switches between the masks during this 4-day period). During this period the patient received NIV at pressure control 14–16 cmH_2_O, PEEP 8–10 cmH_2_O, inspiratory time 0.7 s/ETS 10% at 30 times per minute and a variable FiO_2_ of 0.5–1.0. Mild sedation was reached with intermittent enteral choral hydrate and intravenous dexmedetomidine. The main disadvantage of the total face mask was the relatively high air leak at the top of the mask around the forehead of the patient, which needed regular adjustment. In addition, several nurses reported more discomfort or intolerance of this type of mask, underscoring the need for rotation with the simple anesthetic mask. The main disadvantage of the simple anesthetic mask was the development of pressure-related skin irritation located at the nasal bridge (stage 1 erythema). Both skin pressure injury and degree of air leak were topics occasionally noted in the electronic patient record by the attending nurses, but also actively discussed at the bedside on a daily basis during the patient's stay in the PICU by the members of our ventilator team. Rotation of these two masks led to an acceptable situation with persistent moderate hypercapnia (Table 1) and SpO_2_ above 92% with exception of periods of extensive mucus secretions and plugging. During these moments regular airway suctioning, physiotherapy and adjustments in the position of both masks were necessary. The parents of the patient did not express a clear preference for either of the NIV masks, but were pleased with the ability to rotate them during PICU stay.

**Table 1:** Performance of NIV interfaces.

**Interface**	Mean air leak % (SD)^*^	Mean pH (SD)/pCO2 kPa (SD)^**^	Mean SpO_**2**_/FiO_**2**_ (SD)^*^
Total face mask	41 (15)	7.39 (0.01)/6.65 (0.92)	140 (41)
Simple anesthetic mask	30 (13)	7.43 (0.03)/7.13 (0.56)	165 (54)

* *from 15 random time-point measurements (hourly captured data in the electronic patient data) within a four-day period in the acute phase of disease (first week of PICU admission) during which two NIV masks were rotated at 1-3 times per day;*

** *from daily blood gas analysis within the same four-day period*.

The patient slowly recovered during the next 7–10 days, upon which we initiated short periods of mobilization, while sitting straight up in his own wheel chair. Initially, he was successfully weaned to continuous support on high flow nasal cannula at 1–2 ml/kg/min after 13 days of PICU admission, but presented with recurrence of dyspnea and hypoxemia again necessitating NIV. For persistent bilateral atelectasis on chest X-ray and CT, he received a course of corticosteroids and was switched to ciprofloxacin and clarithromycin based on a positive sputum culture with extended-beta lactam resistant Klebsiella pneumoniae. He finally further recovered with normocapnia and was transferred to the children's hospital general ward on low flow nasal cannula after 32 days of PICU stay. On day 35 after admission he was discharged to home without need for oxygen supplementation.

## Discussion

In this case report, we describe the successful application of pediatric NIV for hypoxic ARF using a simple anesthetic mask with in-house 3D printed quick-release adaptor in a 4-year old boy with cardiofaciocutaneous syndrome. In the last year, this alternative NIV interface has been successfully used in our PICU in patients for which commercially available pediatric NIV masks were unavailable or were associated with an unacceptable air leakage, pressure-related skin injury, and/or patient discomfort. The case illustrates the major challenge related to finding optimal pediatric NIV interfaces, in particular in young children and those with specific facial features as part of a (genetic) syndrome.

The need for personalized NIV masks is currently widely recognized, although potential strategies have mostly focused on long-term use in adults or children in home settings (7–11). Commercially available non-vented pediatric NIV masks are limited in their range of dimensions. NIV mask interfaces were initially developed for adults and then simply reduced in size, resulting in a poor fit for the pediatric population (12). Large variations in the dimensions of faces exist between different pediatric age groups, as well as resulting from underlying (genetic) syndromes. Improper fit of the NIV mask results in patient discomfort and air leaks, which in turn negate the beneficial effect of positive pressure ventilation (3, 4, 11). Modern ventilators using a specific NIV modus, may only compensate for air leakage to a certain degree. In daily practice, nurses often further tighten the mask to create a better seal, however this frequently leads to skin injury even after short-term use. Unfortunately, such interface-related problems are common and often result in failure of NIV therapy with need to escalate to invasive mechanical ventilation.

Among several other groups (7, 8), we are currently developing a research platform to personalize NIV masks using ultrarapid 3D scanning and printing techniques, which may in the future be exploited also in the acute phase of critical illness in the PICU. Wu et al. have reported the development of custom-fitted masks for long-term NIV in adult patients with sleep apnoea or neuromuscular disease using stereolithography 3D printing and magnetic resonance imaging (MRI) data (7). In addition, Willox et al. workers have tested the feasibility of different designs of 3D printed, customized masks in adult volunteers, to inform future development and implementation in pediatric patients (8). Since such personalized treatment strategies have not yet become clinically available, and in particularly not for application in the PICU, in the current case we used an in-house 3D printed adaptor to connect a simple anesthetic mask for its use as an alternative interface for NIV. Such a mask is widely available and about twenty-times lower in costs as compared to commercial NIV masks. The 3D printed adaptor can easily be adapted to any type of anesthetic mask size, is low in production costs (~14€) and is re-usable, which potentially makes it an interesting alternative also for resource limited regions of the world or in times of scarcity of ventilator equipment, as we experienced recently during the beginning of the COVID-19 pandemic.

Although NIV was applied successfully with relatively lower air leakage using this interface in our case, it still needed rotation with another mask to avoid progression of pressure-related skin injury. This was an important limitation of this interface in our current case, and stresses the need for optimal fitted, personalized NIV masks in children for application in the acute, critical illness in addition to the chronic setting. Moreover, as this was a retrospective case report, we have not assessed skin injury in a standardized manner. The skin pressure injury (erythema) was determined and noticed by bedside nurses and physicians who are experienced in working with NIV in children. The air leaks are measured by the ventilator, and thus are objectively assessed. Both skin injury and air leak were topics that were noted in the electronic patient record by the attending nurses, but also actively discussed at the bedside on a daily basis during the patient's stay in the PICU by the members of our ventilator team. Nevertheless, more rigorous studies e.g., with cross-over designs, including more patients are needed to compare the efficiency between different interfaces for pediatric NIV.

In conclusion, we here describe the successful use of an alternative interface using a simple anesthetic mask with in-house 3D printed adaptor for pediatric NIV in a 4-year old boy with cardiofaciocutaneous syndrome admitted to the PICU for hypoxic ARF. Future progress in the field of personalized strategies to optimize NIV masks specifically in young children and those with specific facial features should contribute to reducing failure rates associated with pediatric NIV in the acute phase of critical illness.

## Data Availability Statement

Data concerning the development and production of the 3D printed adaptor and quick-release clips is readily available upon reasonable request.

## Ethics Statement

Written informed consent was obtained from the minor(s)' legal guardian/next of kin for the publication of any potentially identifiable images or data included in this article.

## Author Contributions

RB collected the patient case data and was responsible for the initial version of the manuscript. All authors contributed to revision of the manuscript, and contributed to the initiation and development of the NIV personalization project.

## Conflict of Interest

The authors declare that the research was conducted in the absence of any commercial or financial relationships that could be construed as a potential conflict of interest.

## Publisher's Note

All claims expressed in this article are solely those of the authors and do not necessarily represent those of their affiliated organizations, or those of the publisher, the editors and the reviewers. Any product that may be evaluated in this article, or claim that may be made by its manufacturer, is not guaranteed or endorsed by the publisher.
